# Differences in ocular high order aberrations before and after small incision lenticule extraction for correction of myopia: a systematic review and meta-analysis

**DOI:** 10.3389/fmed.2024.1274101

**Published:** 2024-03-27

**Authors:** Yifan Du, Yu Di, Shan Yang, Fei Mo, Ge Cui, Di Chen, Ying Li

**Affiliations:** Department of Ophthalmology, Peking Union Medical College Hospital, Peking Union Medical College, Chinese Academy of Medical Sciences, Beijing, China

**Keywords:** high order aberrations, small incision lenticule extraction, myopia, spherical aberration, coma aberration, trefoil aberration

## Abstract

**Objective:**

To examine the causes and factors that lead to high order aberration (HOA) during the treatment of myopia using small incision lenticule extraction (SMILE), as well as the differences between SMILE and other corneal refractive surgeries through a systematic review and meta-analysis.

**Methods:**

A systematic search was conducted from January 2015 to February 2023 in Pubmed, Embase, Web of Science, and Google Scholar databases to gather relevant studies on SMILE and HOA. Studies meeting specific criteria were chosen, and clinical data was retrieved for analysis.

**Results:**

This meta-analysis resulted in the inclusion of 19 studies involving 1,503 eyes. Pooled results showed significant induction of total HOA (tHOA, *d* = −0.21, *p* < 0.001), spherical aberration (SA, *d* = −0.11, *p* < 0.001) and coma aberration (CA, *d* = −0.18, *p* < 0.001) after SMILE compared to pre-SMILE, while no significant change in trefoil aberration (TA) was observed (*d* = −0.00, *p* = 0.91). There was a significantly lower induction of tHOA after SMILE compared to femtosecond laser-assisted *in situ* keratomileusis (FS-LASIK, *d* = 0.04, *p* < 0.001), and no significant difference was observed compared to wavefront aberration-guided (WFG) refractive surgery (*d* = 0.00, *p* = 0.75). There was also a significant association between different levels of myopia and astigmatism, duration of follow-up, lenticule thickness, and preoperative central corneal thickness (CCT) on the induction of tHOA after SMILE (*p* < 0.05), while the higher preoperative myopia group (sphere > -5D), lower preoperative astigmatism group (cylinder ≤ -1D), larger lenticule thickness group (lenticule thickness > 100 μm), shorter follow-up group (follow-up 1 month postoperatively) and the thicker CCT group (CCT > 550 μm) brought a significant induction of tHOA compared to the opposite comparison group (*p* < 0.001).

**Conclusion:**

While SMILE can induce HOA significantly, it induces less HOA than FS-LASIK. Postoperative HOA following SMILE can be affected by factors such as myopia, astigmatism, lenticule thickness, CCT, and duration of follow-up. Future research should continue to explore techniques to decrease the induction of HOA by using this methodology.

**Systematic review registration:**

https://www.crd.york.ac.uk/prospero/.

## Introduction

Myopia is one of the most significant public health problems affecting eye health worldwide, especially among adolescents. The World Health Organization (WHO) reports that the overall prevalence of myopia in high-income countries in the Asia-Pacific area is 53.4% of the global population, with East Asia having a similar rate (1). The prevalence of myopia was growing, impacting the quality of life for many individuals. The development of corneal refractive surgeries was addressing this issue (2). Radial keratotomy (RK) was the first corneal refractive surgery developed to correct myopia. Subsequently, photorefractive keratectomy (PRK), laser-assisted *in situ* keratomileusis (LASIK), laser-assisted subepithelial keratomileusis (LASEK), and small incision lenticule extraction (SMILE) were introduced as advancements in the field (3). SMILE, a type of corneal refractive surgery known for its low invasiveness, excellent accuracy, minimal complications, and reduced postoperative corneal irritation symptoms, has received increased attention in recent studies. Sekundo et al. (4) introduced it in 2011. Research on SMILE has shown that it demonstrates strong safety, effectiveness, predictability, and ocular surface stability after surgery. Additionally, it has a low occurrence of postoperative epithelial flap issues and dry eye (5).

Medically generated HOA can raise the likelihood of visual quality problems such as halos, glare, ghost, and reduced contrast sensitivity in corneal refractive surgery (6). Consequently, an increasing number of scholars are concentrating on the postoperative induction of HOA. Wu and Wang (7) discovered that both FS-LASIK and FLEx could cause irregularities in the shape of the corneal surface and a notable induction in postoperative corneal HOA. In contrast, Xia et al. (8) observed that total HOA (tHOA) and vertical coma aberration (CA) were notably elevated after SMILE compared to the preoperative period. Later, certain scholars “customized” the preoperative corneal wavefront aberration which achieved controllability of corneal HOA. The prior occurrence of HOA was eradicated and the development of new HOA was avoided (9). Despite the technical advantages, there is very conflicting data on whether personalized corneal refractive surgery results in decreased postoperative induction of HOA compared to SMILE procedures (10). Kwak et al. (11) discovered that SMILE does not cause spherical aberration (SA) in several types of HOA. However, Zhong et al. (12) observed a notable rise in both SA and CA following SMILE. Currently, SMILE and other corneal refractive surgeries can cause considerable HOA, but there is still uncertainty regarding the specific forms of HOA induced by SMILE and how it affects changes in HOA. Thus, examining, evaluating, and minimizing the occurrence of HOA has become a shared goal for patients and surgeons. This study aims to synthesize data on changes in HOA following SMILE and other corneal refractive surgeries using systematic review and meta-analysis. To examine the development of HOAs caused by SMILE and compare them with other types of corneal refractive surgeries. Finally examining the affecting aspects of HOA, to provide reference and help to alleviate HOA after SMILE.

## Methods

A meta-analysis was conducted following the established procedures of the Meta-analysis of Observational Studies in Epidemiology (MOOSE) (13). The study adhered to the principles outlined in the Declaration of Helsinki and received approval from the Ethics Committee of Peking Union Medical College Hospital (14).

### Search strategy

This study conducted a comprehensive review and meta-analysis of research on SMILE and HOA. Data were retrieved by searching Pubmed, Embase, Web of Science, and Google Scholar databases for all studies on SMILE and HOA published between January 2015 and February 2023, specifically in English. The English search terms used were “small incision lenticule extraction” or “small-incision lenticule extraction” or “SMILE” or “Smile” or “smile” and “high order aberration” or “higher-order aberration” or “high order aberrations” or “HOA” or “HOAs” or “hoa” or “aberration” (the retrieval of different types of HOAs has been included in the above comprehensive retrieval process). The search was conducted across all fields. Articles must be those recently published in the appropriate journal, including papers released online before the print version.

Three ophthalmologists (YiD, YuD and SY) independently conducted the literature screening process and each summarized the chosen papers. Then an experienced ophthalmologist evaluated the literature and identified relevant literature that met the screening criteria, which may be found in the next section on inclusion and exclusion criteria. The screening process is as follows: Literature was summarized and reviewed using Endnote x9 software, then assessed for relevance based on the abstract and title. The remaining literature was fully downloaded and then filtered based on specific inclusion and exclusion criteria to obtain the accessible data.

### Inclusion and exclusion criteria

The study’s inclusion criteria were: (1) observational studies with specific preoperative and postoperative HOA data (retrospective or prospective cohort studies, which require sample selection without specific bias or intervention); (2) surgeries had to be SMILE, but could include other corneal refractive surgeries; (3) patients undergoing surgery had to be myopic with or without varying degrees of astigmatism; (4) articles had to include average, standard deviations, and sample sizes of HOA data that could be extracted or calculated; (5) HOA data had to include tHOA, and other HOAs like SA, CA, or trefoil aberration (TA) if possible; (6) Patients scheduled for surgery must not have any eye conditions other than myopia and should not experience any visual function issues before the operation. The study excluded research that: (1) studies without follow-up data; (2) articles categorized as “review,” “letter,” “commentary,” or “case report”; (3) studies that were published multiple times or used data from the same studies (only one was chosen); (4) studies with incomplete data.

### Data extraction

The study extracted the fundamental data and HOA data from the final conforming literature. The data consisted of author, publication date, sample size, age, aberration analyzer, mean spherical, mean cylinder, scanned pupil diameter, and duration of follow-up. The main observations in this work focused on pre- and post-operative HOA, which included total pre- and post-operative HOA, SA, CA, and TA. The results are presented as the mean difference between these markers (mean ± standard deviation). We also gathered information about other corneal refractive surgeries in case the study involved comparing SMILE with them. We categorize these articles into several subgroups according to factors like spherical, cylinder, duration of follow-up, etc. We next analyze and compare the parameters that influence changes in HOA.

### Quality assessment

We evaluated the quality of the retrospective observational studies included in this analysis using the Newcastle-Ottawa Scale (NOS). Each study’s risk of bias was assessed based on this scale, and the scores for each study were calculated and combined, with scores ranging from 0 (lowest quality) to 9 (highest quality) (15).

### Sensitivity analysis and publication bias

A sensitivity analysis was conducted by performing a “leave-one-out” analysis to assess the robustness of the statistical model. This involved systematically removing each study included in the analysis to examine their impact on the overall pooled estimates. Publication bias was analyzed by Egger’s test (16) and Begg’s test (17) for the included studies, which were determined not to have substantial publication bias if the result was *p* > 0.05.

### Statistical analysis

The study utilized RevMan v5.3 for meta-analysis and to create forest and funnel plots. We utilized *I*^2^ to assess the heterogeneity of the literature included, with a significance level of α = 0.1. Heterogeneity was assessed using the *I*^2^ statistic. If *p* ≥ 0.10 and *I*^2^ ≤ 50%, the studies were deemed homogeneous and a fixed-effects model was used for meta-analysis. Conversely, if *p* < 0.1 and *I*^2^ > 50%, heterogeneity was present, and a random-effects model was employed for meta-analysis. Due to differences in devices, evaluation methodologies, and algorithms among studies, variations in results are inevitable when measuring HOA. While it is challenging to directly compare various devices and algorithms at the individual level, it is still possible to derive HOA values at the group level, albeit the diversity of the research becomes more pronounced. The aberration analyzer utilized in the various investigations has been identified in Table 1. The final observation involves comparing the mean difference between the two groups, where statistical significance is determined by a *p*-value of less than 0.05.

**Table 1:** Summary of basic information on SMILE and HOA related studies included in this study.

Study	Year	Country	Eyes (n)	Mean age	Aberration analyzer	Mean sphere (D)	Mean cylinder (D)	Pupil diameter	Follow-up (mo)	Position	SMILE instrument
Xiaojing Li (18)	2015	China	55	22.22 ± 3.04	Pentacam HR	−5.74 ± 1.39	−0.66 ± 0.70	6 mm	1, 3, 6	Cornea	VisuMax
Jay Jiyong Kwak (11)	2020	South Korea	57	25.59 ± 5.49	iTrace	−4.37 ± 1.98	−1.14 ± 0.82	/	3	Cornea	VisuMax
Ikhyun Jun (19)	2018	South Korea	45	24.80 ± 4.56	Keratron Scout	−3.19 ± 1.55	−2.90 ± 0.42	/	6	Cornea	VisuMax
Manrong Yu (20)	2019	China	64	24.20 ± 4.50	WASCA	−4.10 ± 0.90^*^	/	6 mm	3, 36	Cornea	VisuMax
Hun Lee (21)	2018	South Korea	81	28.30 ± 6.00	Keratron Scout	−4.66 ± 1.25	−1.03 ± 0.75	/	6	Cornea	VisuMax
Ikhyun Jun (22)	2021	South Korea	91	27.79 ± 5.95	Keratron Scout	−3.18 ± 1.28	−0.93 ± 0.69	6 mm	6	Cornea	VisuMax
	2021	South Korea	59	27.32 ± 7.04	Keratron Scout	−3.26 ± 1.49	−0.97 ± 0.94	6 mm	6	Cornea	VisuMax
Yu Zhang (10)	2022	China	102	28.20 ± 6.10	Sirius	−5.09 ± 1.26	−0.63 ± 0.31	6 mm	1, 6	Cornea	VisuMax
*D. rex* Hamilton (23)	2020	America	36	31.60 ± 6.30	Galilei G4	−3.77 ± 1.60	−0.50 ± 0.46	6 mm	1	Cornea	VisuMax
	2020	America	37	29.10 ± 5.10	Galilei G4	−4.02 ± 1.39	−0.10 ± 0.19	6 mm	1	Cornea	VisuMax
Xiaoqin Chen (24)	2017	China	39	22.00 ± 4.00	WaveScan	−4.41 ± 1.23	−2.26 ± 0.73	5 mm	3	Whole eye	VisuMax
Mehmet Gulmez (25)	2020	Turkey	94	27.96 ± 6.43	WaveLight Oculyzer II	−4.89 ± 2.31	−1.64 ± 1.45	6 mm	1, 6	Cornea	VisuMax
Yewei Yin (26)	2021	China	51	23.90 ± 4.80	iTrace	−7.96 ± 0.94^*^	/	4 mm	1, 6, 12	Whole eye	VisuMax
Hong-Ying Jin (27)	2018	China	65	24.46 ± 7.34	Pentacam HR	−6.94 ± 1.00	−0.76 ± 0.68	6 mm	1, 3	Cornea	VisuMax
	2018	China	132	23.84 ± 5.92	Pentacam HR	−4.13 ± 1.00	−0.64 ± 0.50	6 mm	1, 3	Cornea	VisuMax
Wenjing Wu (7)	2016	China	73	24.45 ± 6.30	Pentacam HR	−5.44 ± 1.30	−0.71 ± 0.67	6 mm	3	Cornea	VisuMax
Meiyan Li (28)	2019	China	68	29.50 ± 5.80	Pentacam HR	−5.95 ± 1.37	−0.84 ± 0.78	6 mm	3, 6, 60	Cornea	VisuMax
Yuanyuan Zhong (12)	2020	China	43	24.60 ± 3.90	Pentacam HR	−5.12 ± 1.77	−2.47 ± 0.54	/	48	Cornea	VisuMax
	2020	China	31	25.30 ± 4.20	Pentacam HR	−5.58 ± 1.78	−0.55 ± 0.28	/	48	Cornea	VisuMax
Min-jie Ye (29)	2016	China	170	25.25 ± 4.20	Pentacam HR	−5.03 ± 1.89^*^	/	6 mm	6	Cornea	VisuMax
Fei Xia (8)	2020	China	26	28.27 ± 7.76	Pentacam HR	−5.95 ± 1.14	−0.76 ± 0.48	5 mm	1, 12, 60, 84	Cornea	VisuMax
Weiming Yang (30)	2019	China	29	26.50 ± 7.50	WASCA	−8.23 ± 0.56	−0.94 ± 0.71	6 mm	6	Whole eye	VisuMax
Kang DSY (31)	2018	South Korea	55	28.60 ± 6.40	Keratron Scout	−4.41 ± 1.74	−0.90 ± 0.66	/	3	Cornea	VisuMax

*Spherical equivalent.

## Results

### Results of the screening and sensitivity analysis

**Figure 1:**
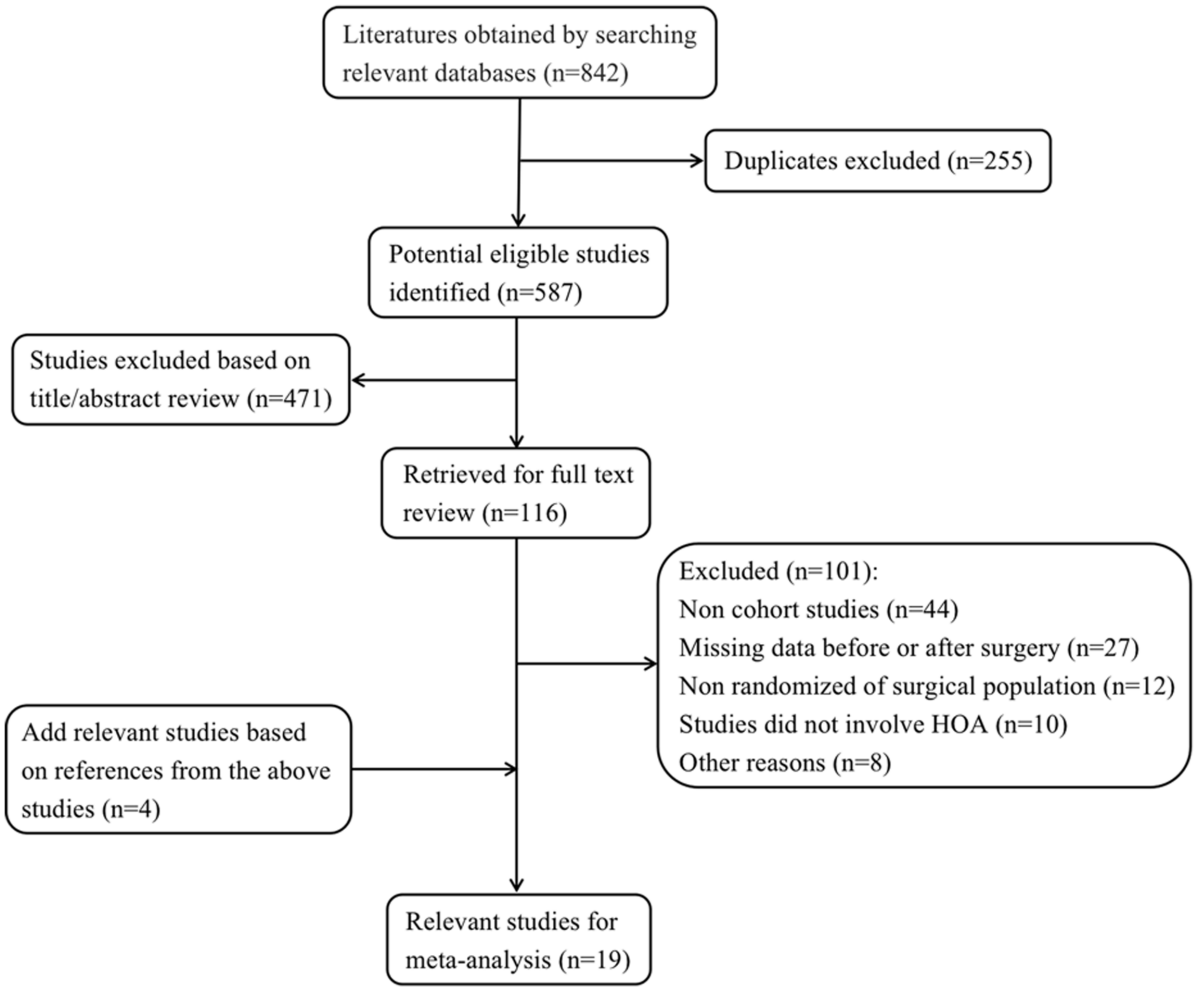
Flow diagram of the literature search.

A total of 842 articles were chosen from the designated search databases based on our search criteria. After eliminating 255 duplicate articles, the remaining 587 articles were initially evaluated based on their study content and focus using titles and abstracts (excluding irrelevant literature). Later, 116 articles were chosen based on the literature screening criteria outlined in the methodology section: Inclusion and Exclusion Criteria. Forty-four articles were not cohort studies; 27 articles did not have pre- or post-operative data; 12 articles did not randomize the surgical population; 10 articles did not include analysis of HOA; and 8 articles were omitted for other reasons. We evaluated the already identified literature and found 4 new articles from their reference sections and cited articles. Ultimately, 19 articles that satisfied our criteria were included in this meta-analysis (7, 8, 10–12, 18–31). Figure 1 displays a flow chart of the literature reviewed in this study. The sensitivity analysis results showed that both the Egger’s test (*p* = 0.178 to 1.000) and Begg’s test (*p* = 0.212 to 1.000) did not reveal any significant publication bias among the 19 included articles. The fundamental details and data from the 19 articles are outlined in Table 1, while the risk of bias and NOS scores for each study are displayed in Table 2.

**Table 2:** Bias risk assessment results and NOS score of included retrospective studies.

Study	Representativeness of the exposed group	Confirmation of no outcome indicators to be observed at the beginning of the study	Identification of exposure factors	Representativeness of the non-exposed group	Comparability of the exposed and unexposed groups was considered in the design and statistical analysis	Evaluation of outcome indicators	Sufficient follow-up time	Completeness of follow-up between the exposed and unexposed groups	NOS score
Xiaojing Li	√	√	√	√	√	√	√	√	8
Jay Jiyong Kwak	√	√	√	√	√	√		√	7
Ikhyun Jun			√	√	√	√	√	√	6
Manrong Yu	√	√	√	√	√	√	√	√	8
Hun Lee	√	√	√	√	√√	√	√	√	9
Ikhyun Jun	√	√	√	√	√	√	√	√	8
Yu Zhang	√	√	√	√	√	√	√	√	8
*D. rex* Hamilton	√	√	√	√	√	√		√	7
Xiaoqin Chen	√	√	√	√	√√	√		√	8
Mehmet Gulmez			√	√	√√	√	√	√	7
Yewei Yin			√	√	√	√	√	√	6
Hong-Ying Jin			√	√	√√	√		√	6
Wenjing Wu	√	√	√	√	√√	√		√	8
Meiyan Li	√	√	√	√	√	√	√	√	8
Yuanyuan Zhong			√	√	√	√	√	√	6
Min-jie Ye	√	√	√	√	√	√	√	√	8
Fei Xia			√	√	√√	√	√	√	7
Weiming Yang			√	√	√	√	√	√	6
Kang DSY	√	√	√	√	√	√		√	7

### Changes in various HOAs after SMILE

**Figure 2:**
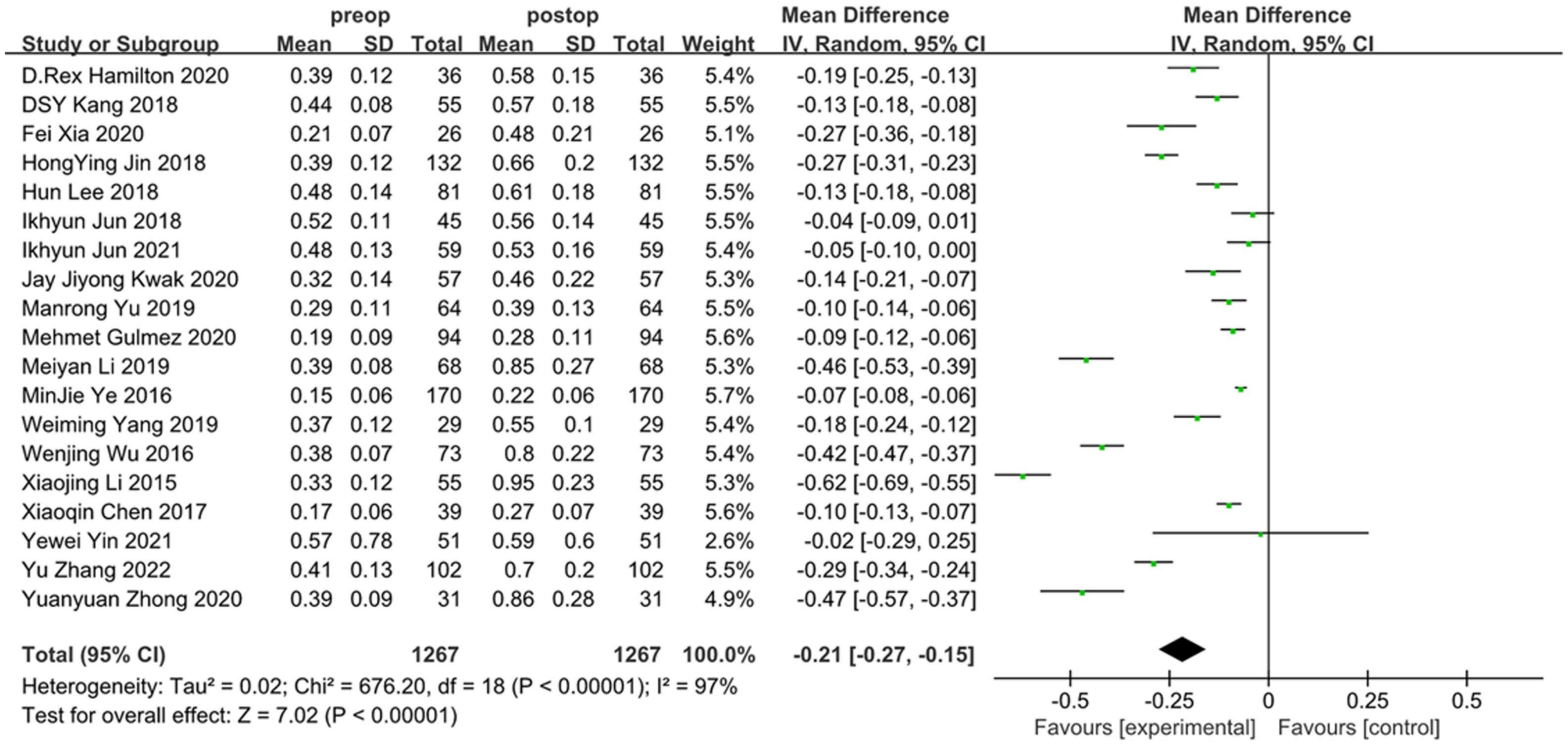
Outcomes of SMILE induced HOAs (tHOA). SD, standard deviation; CI, confidence interval; df, degree(s) of freedom; I^2^, heterogeneity; Z, overall effect.

**Figure 3:**
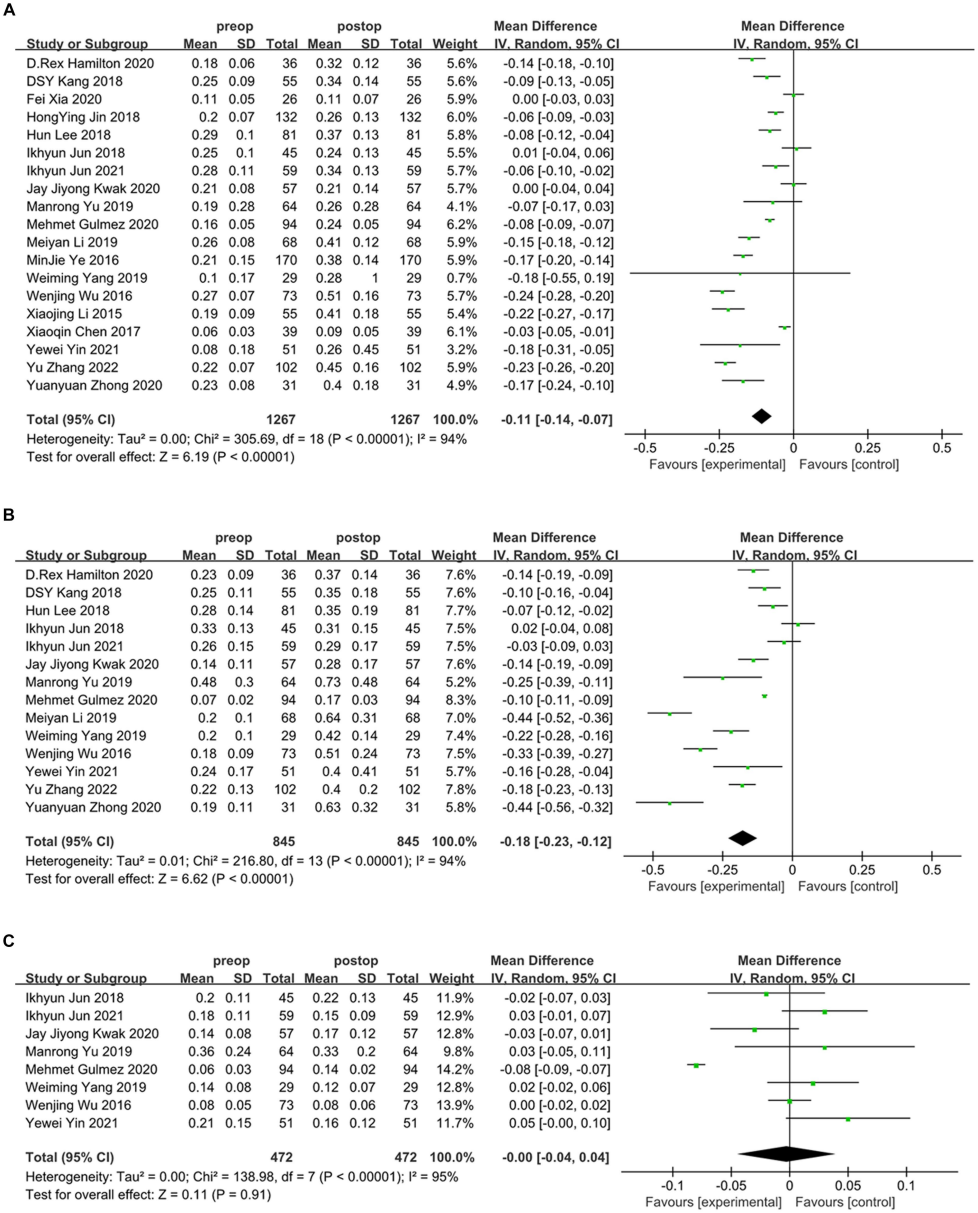
Outcomes of SMILE induced HOAs. **(A)** Spherical aberration introduced by SMILE; **(B)** coma aberration introduced by SMILE; **(C)** trefoil aberration introduced by SMILE. SD, standard deviation; CI, confidence interval; df, degree(s) of freedom; I^2^, heterogeneity; Z, overall effect.

Overall, the results of this meta-analysis showed a significant induction of tHOA (*d* = −0.21, 95%CI: −0.15 to −0.27, *I*^2^ = 97%, random effects model, *p* < 0.001) following SMILE in comparison to before the procedure. When examining each category of HOA individually, SA (*d* = −0.11, 95%CI: −0.07 to −0.14, *I*^2^ = 94%, random effects model, *p* < 0.001) and CA (*d* = −0.18, 95%CI: −0.12 to −0.23, *I*^2^ = 94%, random effects model, *p* < 0.001) were also significantly induced. TA was not substantially affected by SMILE (*d* = −0.00, 95%CI: −0.04 to 0.04, *I*^2^ = 95%, random effects model, *p* = 0.91). The detailed outcomes are displayed in Figures 2, 3 and Table 3.

**Table 3:** Meta-analysis of HOA induction in SMILE and other corneal refractive surgeries.

	Eyes (n)	Mean tHOA differences (95%CI)	Heterogeneity (*I*^2^)	Effects model	Comparison of HOA (*p*-value)
HOAs					
tHOA	1,267	−0.21 (−0.27, −0.15)	97%	Random	**<0.001**
SA	1,267	−0.11 (−0.14, −0.07)	94%	Random	**<0.001**
CA	845	−0.18 (−0.23, −0.12)	94%	Random	**<0.001**
TA	472	−0.00 (−0.04, 0.04)	95%	Random	0.91
Comparison of different surgeries					
FS-LASIK vs. SMILE	356 vs. 482	0.04 (0.02, 0.06)	52%	Fixed	**<0.001**
WFG-surgery vs. SMILE	460 vs. 531	0.00 (−0.01, 0.02)	0%	Fixed	0.75

CA, coma aberration; FS-LASIK, femtosecond laser-assisted in situ keratomileusis; HOA, high order aberration; SA, spherical aberration; SMILE, small incision lenticule extraction; TA, trefoil aberration; tHOA, total high order aberration; WFG, wavefront aberration-guided; Mean tHOA differences are displayed as pre-HOA minus post-HOA and SMILE-HOA minus other-surgery-HOA.*p*-values<0.05 are presented in boldface.

### Postoperative induction of HOA of SMILE versus FS-LASIK and wavefront aberration-guided surgery

**Figure 4:**
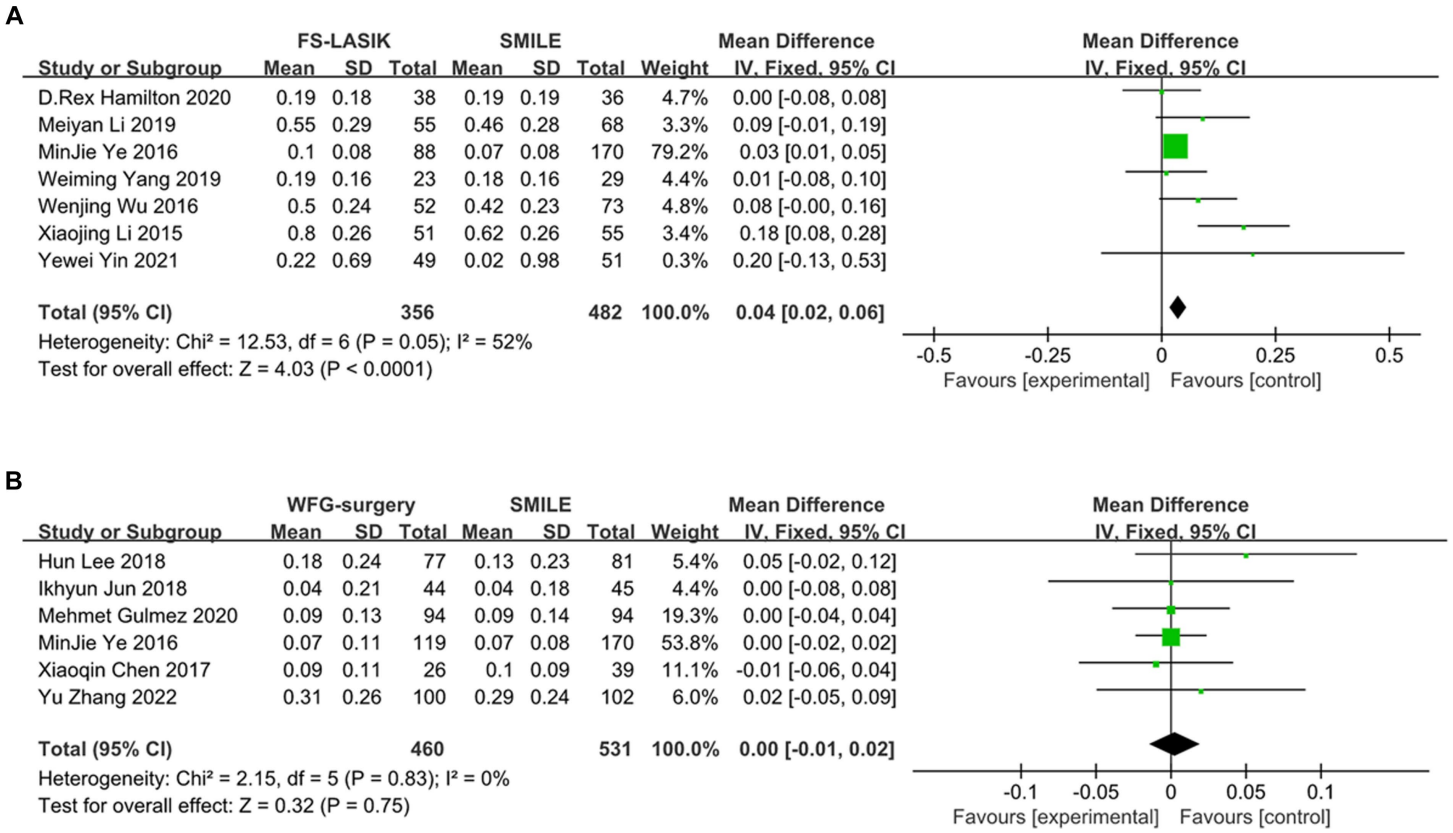
Outcomes of comparison between SMILE and other keratorefractive surgeries. **(A)** SMILE vs. FS-LASIK; **(B)** SMILE vs. WFG-surgery. SD, standard deviation; Cl, confidence interval; df, degree(s) of freedom; l^2^, heterogeneity; Z, overall effect.

The meta-analysis results indicate that SMILE and other corneal refractive surgeries lead to induced HOA compared to FS-LASIK. However, the analysis reveals a significant decrease in induced tHOA after SMILE compared to FS-LASIK (*d* = 0.04, 95%CI: 0.02 to 0.06, *I*^2^ = 52%, fixed-effect model, *p* < 0.001), while there was no significant difference in postoperative induction of tHOA between SMILE and wavefront aberration-guided (WFG) refractive surgery (*d* = 0.00, 95%CI: −0.01 to 0.02, *I*^2^ = 0%, fixed-effect model, *p* = 0.75). The precise outcomes are displayed in Figure 4 and Table 3.

### Factors affecting the change in HOA before and after SMILE

**Figure 5:**
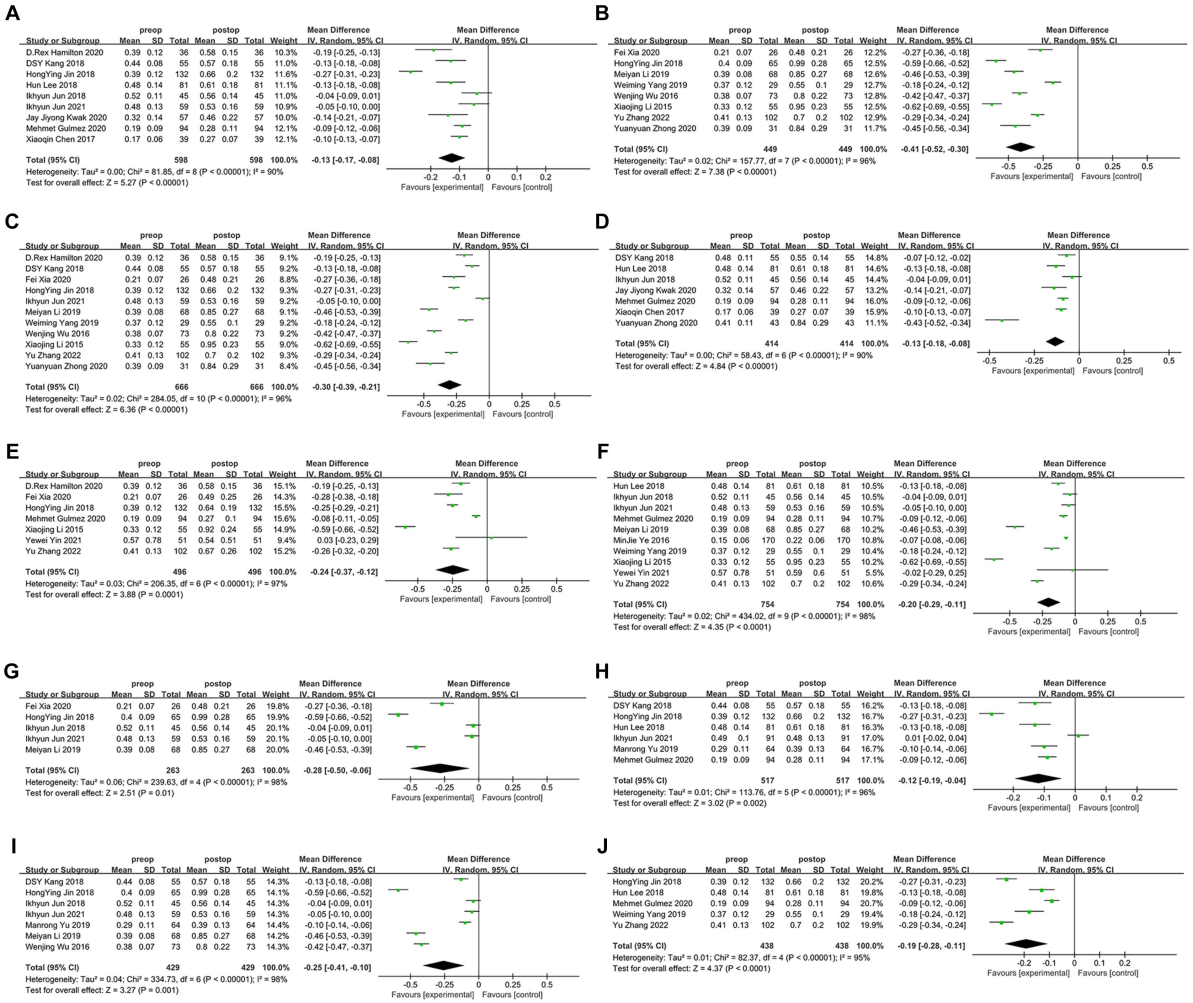
Outcomes of the induction of HOAs by SMILE under different factor grouping. **(A)** The lower myopia (sphere≤-5D); **(B)** the higher myopia group (sphere>-5D); **(C)** the lower astigmatism group (cylinder≤-1D); **(D)** the higher astigmatism group (cylinder>-1D); **(E)** shorter follow-up group (1 month); **(F)** longer follow-up group (6 months); **(G)** thicker lenticule thickness group (>100 μm); **(H)** thinner lenticule thickness group (≤100 μm); **(I)** thicker preoperative CCT group (>550 μm); **(J)** thinner preoperative CCT group (≤550 μm). SD, standard deviation; Cl, confidence interval; df, degree(s) of freedom; l^2^, heterogeneity; Z, overall effect.

After consolidating the data from combining each study, we analyzed the indicators of myopia and astigmatism degree, duration of follow-up, lenticule thickness, and preoperative central corneal thickness (CCT), after which we divided these studies into two groups according to different indicators (whether sphere >-5D, whether cylinder >-1D, follow-up of 1 month and 6 months, whether the lenticule thickness was larger than 100 μm, and whether preoperative CCT was larger than 550 μm). The results showed that there was a significant induction of tHOA after SMILE compared to preoperative for all indicators under the grouping (*p* < 0.05). Analysis by *t*-test showed that the higher myopia group (sphere >-5D, *d* = −0.41, 95%CI: −0.30 to −0.52, *I*^2^ = 96%, random effects model, *p* < 0.001), the lower astigmatism group (cylinder ≤-1D, *d* = −0.30, 95%CI: −0.21 to −0.39, *I*^2^ = 96%, random effects model *p* < 0.001), the group with shorter follow-up (1 month, *d* = −0.24, 95%CI: −0.12 to −0.37, *I*^2^ = 97%, random effects model, *p* < 0.001), the group with greater lenticule thickness (lenticule thickness > 100 μm, *d* = −0.28, 95%CI: −0.06 to −0.50, *I*^2^ = 98%, random effects model, *p* = 0.01) and the thicker preoperative CCT group (preoperative CCT >550 μm, *d* = −0.25, 95%CI: −0.10 to −0.41, *I*^2^ = 98%, random effects model, *p* = 0.001) had significantly induced the postoperative tHOA introduction compared to the comparison group (*p* < 0.05). The precise outcomes are displayed in Figure 5.

## Discussion

The HOA stands for third-order or higher aberrations that are not correctable with conventional eyeglasses or contact lenses. The human eye’s non-flat optical plane leads to uneven refraction and curvature at all sites, which are essential for the development of HOA (32). Although SMILE has accurate cutting powers, it is unable to eliminate the occurrence of HOA. The meta-analysis results indicate a statistically significant induction of HOAs after SMILE compared to before the procedure, suggesting that SMILE can lead to a considerable induction of HOAs. The outcome was consistent in most research. Jun et al. (19) and Yin et al. (26) did not observe a significant induction of tHOA following SMILE in their trials. After reviewing the manuscripts from Yin et al. (26), we observed that the mean preoperative spherical equivalent (SE) of the study population was high (>-7D). However, the pupil diameter analyzed was only 4 mm, with most being 6 mm. It indicates that the induction of HOA in the central vision region may not be significant. Consequently, the decreased visual quality likely occurred more frequently at night following SMILE. Jun et al. (19) utilized a trifocal focus method for SMILE with a mean preoperative SE of approximately -3D, potentially accounting for the little induction of tHOA following their SMILE treatment.

Most studies on various forms of HOA have shown a considerable induction of SA after SMILE, and our meta-analysis confirmed these findings. Several investigations have shown varying results on the induction of SA after SMILE, possibly due to changes in instrumentation, dark-vision environment, and pupil size (33, 34). Kwak et al. (11) and Jun et al. (19) corroborated this using a trifocal focusing approach to decrease the induction of SA. Xia et al. (8) did not see a significant induction of SA, possibly because there were too few corneal areas analyzed (5 mm). Yin et al. (26) observed a notable induction of SA in the 4 mm corneal analysis areas, possibly due to severe myopia (−7.96 ± 0.94D). Greater myopia requires a thicker stromal lenticule to be sliced, leading to significant alterations in the cornea’s front surface and increased induction of SA. The femtosecond laser enables precise excision of corneal tissue, reducing the induction of SA compared to previous surgical methods (35). CA has also been found to be greatly introduced following SMILE, comparable to SA. This meta-analysis yielded consistent findings, except Jun et al. (19), who employed the trifocal focusing approach to minimize the induction of *CA.* Some studies suggest that a single incision during SMILE may result in an uneven corneal healing response, causing optical alterations that can lead to increased induction of CA in the postoperative period (36). Previous research has indicated that reducing eccentricity and promoting wound healing may affect the induction of CA (18). Li et al. (28) showed that horizontal eccentricity leads to horizontal CA induction, while there seems to be no connection between the extent of vertical eccentricity and vertical CA induction. More research is required to explore the origin of both vertical and horizontal CA to enhance the clarity of postoperative visual acuity. This meta-analysis did not discover any significant induction of TA caused by SMILE. Due to the absence of studies directly examining the relationship between TA and SMILE, we explored research on FS-LASIK. Kim et al. (37) discovered that HOAs changed after creating a flap in FS-LASIK, leading to the induction of CA and TA. They proposed that the location of the flap hinge and the microkeratome type could impact the type of aberrations induced during flap creation. The manifestation of TA induction, consistent with the incision location, is attributed to the biomechanical response that occurs after creating the corneal incision. This response involves the steepening of the corneal curvature near the flap hinge and flattening toward the free edge of the flap post-flap creation. SMILE utilizes a femtosecond laser to induce two distinct depth interlayer explosions within the cornea, followed by the creation of a tiny incision measuring 2-4 mm using the same laser. The corneal lenticule is extracted from the tiny incision. FS-LASIK involves creating a 20 mm corneal flap at approximately 270 degrees with a femtosecond laser, followed by cutting the corneal stroma behind the flap with an excimer laser. The reason for the minimal change in TA following SMILE is due to the smaller corneal incision or flap, indicating a distinct benefit in decreasing the induction of TA with SMILE (Table 3).

We examined the variations in the induction of HOA between SMILE and other types of corneal refractive surgeries in this meta-analysis. Comparing SMILE with FS-LASIK, the trials yielded diverse findings, but the combined outcome indicated that SMILE resulted in reduced induction of tHOA. Hamilton et al. (23) did not detect a difference between the techniques due to the use of a wavefront aberration optimization technique for FS-LASIK, which is slightly different from the WFG technique. Yang et al. (30) conducted research that yielded comparable results, which they attributed to the inclusion of participants with high myopia. The high degree of myopia itself dominated the postoperative HOA changes, leading to the fact that the different surgical modalities did not play a key role in this. Ganesh and Gupta (38) discovered no statistically significant difference between the SMILE and FS-LASIK groups in terms of HOA, SA, and CA induced at 6 months after correcting high myopia. This supports the speculation mentioned above, but more research is required to investigate the precise reasons. Contrary findings have been proposed by Li et al. (18) and Yin et al. (26), indicating that SMILE-induced HOA was notably lower than those of FS-LASIK (Table 3).

While LASIK and PRK use pupil-tracking devices to centrally focus on the cornea, SMILE requires the surgeon to manually center the instrument, usually with the corneal vertex as the target. According to the evidence that is now available, SA induction and subsequent tHOA induction both increased as the effective optical zone decentration from the corneal vertex did when SMILE used these centralized approaches (39). The outcomes of PRK and LASIK were also comparable. There was a robust relationship between the degree of vertical decentration and the degree of vertical CA induction. After reviewing the literature on WFG technology and comparing WFG refractive surgery with SMILE in this meta-analysis, we discovered that there was no significant difference in the induction of tHOA between the two procedures postoperatively. Also, while comparing WFG-PRK with SMILE, Jun et al. (19) discovered no statistically significant difference in corneal tHOA before or after surgery. This might be associated with the fact that SMILE makes use of the trifocal focusing approach. We next looked into the literature to see if SMILE had any influence on other kinds of HOA; we discovered that Ganesh and Gupta (38), Liu et al. (40), and Lin et al. (41) had previously found that SMILE-induced SA was much lower than WFG FS-LASIK. Rather than using corneal tissue photodetachment to create microlensing, they proposed that femtosecond laser-assisted tissue stripping could be responsible for the minimal SA induction in SMILE. Because astigmatism correction creates an elliptical posterior surface of the microlens, the optical surface’s diameter is smaller along the steep axis compared to the flat axis; as a result, Ye et al. (29) speculated that this might explain why SMILE caused more horizontal and vertical CA than WFG-LASIK. In addition, the vertical edge of the refractive microlens in SMILE may also contribute to coma compared with the 2.0 mm transition zone around the optical zone in WFG-LASIK. Related to the aforementioned results, Gulmez et al. (25) and Gyldenkerne et al. (42) discovered that SMILE caused more vertical CA and TA than WFG FS-LASIK, and that WFG FS-LASIK caused more SA than SMILE. So there are still significant differences between the various types of HOAs, even if there was no change in the induction of tHOA. Corneal refractive surgery guided by corneal topography is also one of the most popular ways now utilized, both SMILE and its approaches have proven to be effective and safe, according to FDA investigations. However, which technique is better is up for debate because different patients with varied corneal characteristics, topographic irregularities, or HOAs have shown advantages and disadvantages with each treatment (43). Several prospective studies have shown that topography-guided LASIK (TG-LASIK) reduces ocular TA, corneal tHOA, and CA, and causes fewer HOAs overall (44). To achieve the best possible corneal curvature, TG-LASIK uses measurements of the cornea’s topography to guide a personalized ablation procedure. In TG-LASIK, unlike wavefront measurements, the corneal curvature may be measured regardless of the size of the pupil. Correction of peripheral corneal abnormalities on the cornea, where most HOAs of the optical system of the eye emerge, is possible using topography-guided bespoke ablation treatment, and it is not affected by mistakes caused by the pupil centroid shift when the pupil changes size (45, 46). When comparing SMILE and other methods, Yang et al. (47) discovered that SMILE successfully reduced surgically caused HOAs by adjusting for the kappa angle using topography as a reference. When it comes to correcting astigmatism and preoperative anterior corneal hyperopia, TG-LASIK with iris recognition and cyclotorsional adjustment is the way to go.

This meta-analysis investigated factors that could affect the induction of HOA following SMILE. This meta-analysis discovered that the induction of postoperative tHOA was notably greater in the high myopia group (sphere >-5D) compared to the low myopia group (sphere ≤-5D). Just as Jin et al. (27) found that the corneal tHOA, notably vertical CA and SA, were connected to the SE. These results are comparable to the study by Wu and Wang (7), who stated that more investigations are needed to determine the etiology of vertical and horizontal CA to optimize postoperative visual quality. We hypothesize that this effect should be related to the fact that the higher the degree of myopia, the thicker the lens would be cut, thus leading to a stronger induction of postoperative HOA. In contrast, the effect of astigmatism was inverted, and this meta-analysis indicated that the induction of tHOA by SMILE was substantially higher in the group with lower astigmatism (cylinder ≤-1D) than in the group with higher astigmatism (cylinder >-1D). We investigated relevant literature and re-examined the original data to discover the cause. We showed that although SMILE obtained a circular optical zone in the treatment of myopia and astigmatism, astigmatism specifically achieved an elliptical functional optical zone (FOZ). Liu et al. (48) validated in their investigation that eyes with significant astigmatism achieved a higher FOZ and lesser SA induction following SMILE compared to eyes without astigmatism. Therefore, they believe that increasing the size of FOZ can lessen the influence of eccentricity on HOA induction. Recently, Moshirfar et al. (39) have hypothesized that SMILE produces a larger FOZ than LASIK and PRK, which is crucial to minimizing HOA induction. Therefore, they argue that ensuring sufficient FOZ can reduce HOA induction, including SA and vertical CA. This may be the reason why increased astigmatism induces less HOA. In addition, we also analyzed other relevant characteristics of these studies and discovered that the average myopia degree in the high astigmatism group (mean sphere = −4.35 ± 0.66D) was lower than that in the low astigmatism group (mean sphere = −5.23 ± 1.36D). According to the earlier analysis of myopia degree, this will lead to reduced HOA induction in the high astigmatism group due to low myopia degree. However, we observed that there was no significant difference in the degree of myopia between these two groups (*p* = 0.13), thus we suggest that the difference in myopia may be involved in confusing the results, but it is not a crucial issue. Therefore, we believe that the key to the influence of astigmatism on HOA induction is still the magnitude of FOZ, which may indicate that larger astigmatism has less HOA induction. Additional study is required to validate this finding with improved control over pertinent variables.

We also found a substantial tHOA induced in the 1-month follow-up group following SMILE compared to the 6-month follow-up group in this meta-analysis, consistent with findings from studies with longer follow-up periods. According to Pedersen et al. (49), CA remained constant, whereas less SA and tHOA were induced significantly from 3 months to 3 years after SMILE. Most studies have consistently reported enduring stability in HOA following SMILE. Xia et al. (8) discovered that levels of tHOA, SA, CA, and TA remained consistent at specific postoperative time intervals following SMILE, aligning closely with findings from studies by Li et al. (18), Lin et al. (41), and Ağca et al. (50). For the lenticule thickness, Wallerstein et al. (51) verified that in FS-LASIK, the postoperative HOA was considerably higher in the group with a lenticule thickness larger than 100 μm compared to the group with a lenticule thickness less than 100 μm. We believe that increasing the depth of ablation will reduce the precision of ablation from SMILE, and the refractive difference induced by the ablation edge will also be more significant. Eyes with greater lens thickness tend to have a reduced residual corneal bed and corneal thickness, leading to less biomechanical stability and increased induction of HOA. It is logical to assume that the mentioned causes can result in this contrasted outcome, and the connection between myopia severity and the alteration in HOA before and after surgery is somewhat influenced by this. Our study also demonstrated that the SMILE preoperative CCT above 550 μm group produced significantly higher postoperative HOA than the preoperative CCT below 550 μm group. Feng et al. (52) suggested that keeping a larger residual CCT (preoperative CCT) with FS-LASIK can result in reduced SA, despite the absence of specific analysis. They hypothesize that alterations in corneal shape can result in biomechanical modifications, suggesting that HOA could be associated with postoperative biomechanical changes following FS-LASIK. Yet, the evaluation of SMILE appears to be more consistent, as a decrease in corneal thickness does not necessarily result in increased induction of postoperative HOA. Mohamed et al. (53) found no link between central/peripheral corneal thickness and HOA in the eye and cornea. Qu et al. (54) discovered that just one Zernike aberration, Z42, was significantly linked to CCT in corneal HOA. We believe that the results require validation from more investigations. There is also a link between myopia, lenticule thickness, and CCT. A higher degree of myopia requires a thicker lenticule to be sliced, resulting in a thinner postoperative CCT. The correlation between these parameters and HOA is linked to the biomechanical stability of the cornea. Previous studies have shown that the biomechanical stability of the cornea is significantly correlated with the 3rd to 6th-order HOAs and SA induced on the anterior surface and total cornea after SMILE (55). We hypothesize that the relationship between these factors and HOA may be attributed to corneal biomechanics. Future studies in this field will help clarify their direct association and influence (Table 4).

**Table 4:** Meta-analysis and comparative results of influencing factors on HOA changes after SMILE.

Characteristics	Eyes (n)	Mean tHOA differences (95%CI)	Heterogeneity (*I*^2^)	Effects model	Comparison of preop and postop tHOA (*p-*value)	Comparison of subgroup tHOA (*p*-value)
Myopia						
Sphere≤-5D	598	−0.13 (−0.17, −0.08)	90%	Random	<0.001	<0.001
Sphere>-5D	449	−0.41 (−0.52, −0.30)	96%	Random	<0.001	
Astigmatism						
Cylinder≤-1D	666	−0.30 (−0.39, −0.21)	96%	Random	<0.001	<0.001
Cylinder>-1D	414	−0.13 (−0.18, −0.08)	90%	Random	<0.001	
Follow-up time						
1 month	496	−0.24 (−0.37, −0.12)	97%	Random	<0.001	<0.001
6 months	754	−0.20 (−0.29, −0.11)	98%	Random	<0.001	
Preop CCT						
>550 μm	429	−0.25 (−0.41, −0.10)	98%	Random	=0.001	<0.001
≤550 μm	438	−0.19 (−0.28, −0.11)	95%	Random	<0.001	
Lenticule thickness						
>100 μm	263	−0.28 (−0.50, −0.06)	98%	Random	=0.01	<0.001
≤100 μm	517	−0.12 (−0.19, −0.04)	96%	Random	=0.002	

CCT, central corneal thickness; HOA, high order aberration; SMILE, small incision lenticule extraction; tHOA, total high order aberration; Mean tHOA differences are displayed as pre-HOA minus post-HOA.*P-*values<0.05 are presented in boldface.

There are some limitations of our study: (1) Due to the diverse origins of these studies, variations in aberration analyzers, and differences in populations, the results of aberration measurements and analyses may be somewhat influenced. However, this study primarily focused on comparing HOA before and after SMILE, with no changes observed in these factors before and after the procedure. Hence, the impact of these differences can be mostly disregarded. (2) Most studies use a 6 mm pupil size for analyzing HOA, but a few studies use a smaller diameter of 5 mm or less (some do not specify). Detecting HOA changes becomes challenging with a small pupil size, which can influence the disparity in HOA before and after surgery. (3) Ocular HOA mostly originates from the cornea but also involves refractive components such as the lens, anterior chamber, and vitreous. Only a few studies in this meta-analysis focused on whole-eye HOA, which may have partially impacted the accuracy. However, as SMILE is a type of corneal refractive surgery that only alters the cornea, this issue has minimal influence. (4) Discrepancies in the postoperative duration of follow-up may result in alterations in HOA, so selecting varying time intervals following the surgery can provide diverse outcomes. The study attempted to use a uniform postoperative follow-up period of 6 months for analysis, but this variation still impacted the results of the analysis. (5) The choice of cutting area, energy level, and corneal cap thickness in SMILE can impact the variations in HOA. This study aims to choose coherent studies for analysis, while minor distinctions may still impact the final result. Other factors such as selection bias and publication bias may also have some impact on this meta-analysis.

## Conclusion

According to this analysis and previous studies, SMILE can dramatically increase the induction of tHOA, SA, and CA, but not TA. SMILE can greatly decrease the induction of tHOA compared to FS-LASIK, and there is no notable difference in tHOA generated by WFG refractive surgery. After categorizing by various factors, the group with severe myopia (sphere >-5D), lower astigmatism (cylinder ≤-1D), shorter duration of postoperative follow-up (1 month), thicker lenticule (over 100 μm), and higher preoperative CCT (over 550 μm) exhibited increased induction of tHOA compared to their respective control groups during SMILE. Despite the inevitable induction of HOA, SMILE remains a safe and successful procedure for corneal refractive surgery, particularly in minimizing the induction of tHOA, SA, and CA. When choosing surgical techniques in clinical practice, it is important to examine issues such as safety, effectiveness, postoperative complications, and the potential induction of postoperative HOA. To some extent, ensuring the stability of visual quality in myopic populations can be achieved by minimizing the occurrence of postoperative HOA during surgery.

## Data availability statement

The original contributions presented in the study are included in the article/supplementary material, further inquiries can be directed to the corresponding author.

## Author contributions

YiD: Writing – review & editing, Writing – original draft, Methodology, Investigation, Funding acquisition, Formal analysis, Data curation, Conceptualization. YuD: Writing – original draft, Investigation, Data curation. SY: Writing – original draft, Resources, Formal analysis. FM: Writing – original draft, Software, Methodology. GC: Writing – original draft, Resources. DC: Writing – review & editing, Visualization, Validation. YL: Writing – review & editing, Visualization, Validation, Supervision, Methodology, Funding acquisition.
